# The Doses and Actions of Medicines

**Published:** 1868-07

**Authors:** 


### The Doses and Actions of Medicines.

He considered that the prevalent distrust in the efficacy of drugs might all be resolved into the three following formulæ:—1. The natural reaction from the overweening confidence in medicine, and from the wholesale and indiscriminate administration of drugs which prevailed at the beginning of the present century. 2. The discovery that certain disorders, if left to themselves, in many instances tend to recovery. 3. The prevalence of extreme ignorance as to the dose in which each drug is tolerated by the system, and as to the dose in which it must be administered in order to obtain its curative effect. After enlarging somewhat on these points, Dr. Fuller concluded an able paper by remarking that by one means or another distrust had come to prevail, and discredit had been thrown on the curative action of drugs. Disease was left to take its natural course, and homœopathy was practiced under the guise of scientific medicine. The public, who see by the reports of cases in our periodical literature how little heed some men pay to medicine, were becoming indoctrinated with the belief that drugs are of no avail, and were consistently betaking themselves to the professed homœopath. For this belief Dr. Fuller maintained that there were absolutely no grounds, and urged the Society to appoint a committee for the purpose of investigating the subject of therapeutics and the action of medicine. If, he said, it should prove, on inquiry, that drugs are of no avail in modifying the course of disease, let us, as honest men, avow our mistake, alter our practice, and admit that homœopaths have had just cause for their vilification of the legitimate practice of medicine; but if, on the contrary, the labors of the committee resulted in establishing the curative action of medicines, where properly administered in doses and in combinations suited to the exigencies of each case, the profession might fairly hope to obtain as the result of the investigation some trustworthy therapeutical data. The investigation must necessarily be coeval with the existence of the healing art; but the labors of the committee, if carefully conducted, would serve as a model to future inquirers; the conclusions at which they arrived would be a nucleous to which other facts might be added from time to time, until information was obtained in the healing art which would be indispensable to every practitioner; and meanwhile the Harveian Society would have the honor of inaugurating an inquiry which would not have been unworthy of the Royal College of Physicians.—*Lancet.*

